# Whole tumor antigen vaccination using dendritic cells: Comparison of RNA electroporation and pulsing with UV-irradiated tumor cells

**DOI:** 10.1186/1479-5876-6-21

**Published:** 2008-04-29

**Authors:** Fabian Benencia, Maria C Courrèges, George Coukos

**Affiliations:** 1 Center for Research on Early Detection and Cure of Ovarian Cancer, University of Pennsylvania, Philadelphia, PA, USA; 2 Abramson Family Cancer Research Institute, University of Pennsylvania, Philadelphia, PA, USA; 3 Department of Biomedical Sciences, College of Osteopathic Medicine and Biomedical Engineering Program, Russ College of Engineering and Technology, University of Ohio, Athens, OH, USA

## Abstract

Because of the lack of full characterization of tumor associated antigens for solid tumors, whole antigen use is a convenient approach to tumor vaccination. Tumor RNA and apoptotic tumor cells have been used as a source of whole tumor antigen to prepare dendritic cell (DC) based tumor vaccines, but their efficacy has not been directly compared. Here we compare directly RNA electroporation and pulsing of DCs with whole tumor cells killed by ultraviolet (UV) B radiation using a convenient tumor model expressing human papilloma virus (HPV) E6 and E7 oncogenes. Although both approaches led to DCs presenting tumor antigen, electroporation with tumor cell total RNA induced a significantly higher frequency of tumor-reactive IFN-gamma secreting T cells, and E7-specific CD8+ lymphocytes compared to pulsing with UV-irradiated tumor cells. DCs electroporated with tumor cell RNA induced a larger tumor infiltration by T cells and produced a significantly stronger delay in tumor growth compared to DCs pulsed with UV-irradiated tumor cells. We conclude that electroporation with whole tumor cell RNA and pulsing with UV-irradiated tumor cells are both effective in eliciting antitumor immune response, but RNA electroporation results in more potent tumor vaccination under the examined experimental conditions.

## Introduction

Because tumor-associated antigens are not well characterized for the majority of human tumors, polyvalent vaccines prepared with whole tumor antigen are an attractive approach to induce tumor vaccination [1,2]. Recent advances in generation and manipulation of DCs provide opportunities to design powerful tumor vaccines. DCs are ideal vehicles for polyvalent tumor vaccination, as they readily process and present tumor antigen taken up from dying tumor cells.

DCs pulsed with apoptotic tumor cells have been used successfully to induce tumor vaccination [3-12]. Although controversy surrounds the ability of necrotic versus apoptotic tumor cells to serve as a source of multivalent antigen to pulse DCs [10,13-15], UVB irradiation has been shown to result in a mixed population of apoptotic and necrotic tumor cells [16]. Tumor cells exposed to lethal ultraviolet-B (UVB) radiation have been shown to provide a suitable source of tumor antigen for DCs [16,17]. For example, UV-irradiated primary tumor cells provide sufficient tumor antigen to elicit expansion of tumor-reactive autologous T cells ex vivo in patients with advanced ovarian cancer [17], suggesting that this approach can be used clinically to induce therapeutic vaccination.

Several reports have described the use of tumor-extracted RNA as source of tumor antigen for the preparation of DCs and have indicated its potential use for antigen-specific or polyvalent tumor vaccination in the absence of identified tumor antigens [18-23]. Such approach may address important limitations in the procurement of tumor antigen, as primary tumor cell cultures are not feasible for a large number of patients. Although the feasibility and efficacy of electroporation of DCs with RNA for the preparation of polyvalent tumor vaccines has been convincingly demonstrated, a direct comparison of DC vaccines prepared with tumor RNA versus dying whole tumor cells has not been performed.

In this study, we compared tumor RNA to apoptotic tumor cells as a source of tumor antigen to generate a DC-based vaccine against tumors expressing the early gene products E6 and E7 of the human papilloma virus (HPV). We report that the use of tumor RNA as a source of tumor antigen is valuable alternative and superior to UV-irradiated tumor cells.

## Materials and methods

### Cell lines

TC-1, cell line was a generous gift from Dr. Yvonne Paterson, University of Pennsylvania. TC-1 cells were maintained in RPMI (Invitrogen, Carlsbad, CA) supplemented with L-glutamine (2 mM); penicillin (100 U/ml); streptomycin (100 μg/ml); 10% fetal bovine serum (FBS), and geneticin (1 mg/ml).

ID8, a cell line derived from spontaneous in vitro malignant transformation of C57BL/6 mouse ovarian surface epithelial cells, was a generous gift from Dr. Paul F. Terranova, University of Kansas [24]. ID8 cells were maintained in DMEM medium (Invitrogen) supplemented with 4% FBS, penicillin, streptomycin, insulin (5 μg/ml), transferrin (5 μg/ml), and sodium selenite (5 ng/ml, all Roche, Indianapolis, IN) in a 5% CO_2 _atmosphere at 37°C. An ID8 cell line expressing the HPV16 E6 and E7 antigens was generated by transducing ID8 cells with the retroviral vector LXSN16E6E7 (American Type Culture Collection, Rockville, MD, donated by Dr. D. Galloway), which encodes the HPV16 E6 and E7 genes, as well as the neomycin phosphotransferase gene. The PA317 cell line was used to generate the retroviral vectors as previously described [25]. Selection of ID8 cells transduced with E6 and E7 (ID8-E6/7) or ID8 cells transduced with a control retroviral vector (LXSN) was achieved under neomycin pressure (1 mg/ml) [25]. The murine L929 (ATCC) immortalized cell line was grown in RPMI 1640 with 10% FBS and penicillin/streptomycin. All lines tested negative for Mycoplasma by PCR.

### Animals and tumors

Six to eight week old female C57BL/6 (H-2K^b^) and BALB/c (H-2K^d^) mice (Charles River Laboratories, Wilmington, MA) were used in protocols approved by the Institutional Animal Care and Use Committee and the University of Pennsylvania. TC-1 tumors were generated in C57BL/6 mice by s.c. inoculation of 2 × 10^4 ^TC-1 cells in 0.2 ml of PBS. Tumors were detectable ten days later and were measured weekly using a Vernier caliper. Tumor volumes were calculated by the formula V= 1/2 L × W^2^, where L is length (longest dimension) and W is width (shortest dimension). For some in vivo studies, CD8^+ ^cells were depleted with rat anti-mouse CD8 (MCA1768XZ) and CD4^+ ^cells with rat anti-mouse CD4 (MCA1767XZ) (both Serotec, Raleigh, NC). The antibodies were administered intravenously (100 μg/animal) on the day of tumor injection and a second dose one week later.

### Generation of bone marrow-derived DCs

Murine dendritic cells were generated from bone marrow precursor cells with recombinant murine granulocyte-macrophage colony-stimulating factor (GM-CSF; Peprotech, Rocky Hill, NJ; 20 ng/ml) as described previously [26]. Cells were counted using Trypan blue. Differentiation into immature DCs was documented through flow cytometry detection of CD80, CD86 and major histocompatibility complex class II (MHC-II). DC maturation was induced by culturing cells in RPMI media under standard conditions in the presence of 10 ng/ml murine GM-CSF supplemented with 0.1 μg/ml lipopolysaccharide (LPS, Sigma Chemical Co, Saint Louis, MO) and 20 ng/ml tumor necrosis factor-alpha (TNF-α, Peprotech).

### DC electroporation with tumor RNA

Total cellular RNA was extracted from TC-1 cells using TRIzol Reagent (Invitrogen). Cells grown in 75 cm^2 ^flasks were resuspended and lysed using TRIzol reagent. Chloroform (0.2 ml per ml of TRIzol reagent) was added and incubated at room temperature for 2 min. The samples were centrifuged at 12,000 × g for 15 min at 4°C, and the aqueous phase was transferred to a new tube. Cold isopropanol was added at 0.5 ml per ml TRIzol reagent to precipitate RNA. Following 10 min incubation at room temperature the samples were centrifuged at 12,000 × g for 10 min at 4°C. The RNA pellet was washed once with 70% DEPC-ethanol and centrifuged at 7500 × g for 5 min. The pellet was briefly dried and dissolved in DEPC water. The quality and quantity of the total RNA was checked using RNA Nano LabChip^® ^(Agilent Technologies, Palo Alto, CA) according to the protocol provided. Two million DCs were resuspended with the appropriate amount of total TC-1 RNA in a 0.2-cm cuvette and electroporated using Gene Pulser II (BIO-RAD Laboratories, Hercules, CA) under different voltage and capacitance settings. DCs electroporated in the absence of TC-1 RNA (mock) were used as controls for some experiments.

### DC pulsing with apoptotic tumor cells

Subconfluent cultures of TC-1 cells were rinsed twice in phosphate buffer saline (PBS) and exposed to ultraviolet-B (UVB) radiation at various doses up to 1500 μW/cm^2 ^for various times. Apoptosis at 24 hours was quantified by flow cytometry detection of annexin-V staining using the TACS™ Annexin-Biotin Apoptosis detection kit (R&D Systems, Minneapolis, MN) and confirmed with the ApopTag peroxidase *in situ *detection kit (Intergen, Purchase, NY) and the Apoptotic DNA-Ladder Kit (Roche), according to the manufacturers' instructions. Twenty-four hours after UVB radiation tumor cells were incubated with immature DCs at a 2:1 ratio (tumor cells, DCs). Twenty-four hours later, TNF-α (Peprotech; 20 ng/ml) and LPS (0.1 μg/ml) were added for additional 48 hours. DCs were harvested, rinsed and counted by trypan blue exclusion. In some experiments, radiated tumor cells were labeled with PKH26 fluorescent dye (Sigma; 5 μM) for 5 min at room temperature. RPMI supplemented with 10% FBS was added to stop the reaction and cells were rinsed three times prior to using them for pulsing DCs.

### Animal vaccination

Animals received one intraperitoneally (i.p.) and then twice subcutaneously (s.c.) seven days apart, DCs (5 × 10^5 ^per dose) electroporated with TC-1 RNA or loaded with TC-1 cells killed by UVB and incubated with TNF-α (20 ng/ml) and LPS (0.1 μg/ml) were injected once. DCs. Control animals were injected with DCs mock electroporated and matured with TNF-α and LPS. Animals were challenged with tumor cells seven days after the last DC vaccination.

### Flow cytometry

Cells were subjected to four-color flow cytometry on a FACSCalibur flow cytometer using CellQuest 3.2.1f1 software (Becton Dickinson, San Jose, CA). Non-specific staining was blocked with anti-CD16/CD32 antibody (Fc block, 2.4G2; BD Pharmingen; San Diego, CA). Fluorochrome-conjugated monoclonal antibodies against CD3 (17A2), CD8 [53–67], CD11c (HL3), CD80 (16-10A1), CD86 (GL1), MHC-I (H-2 kb/H-2Db), MHC-II (KH74; all BD Pharmingen, San Diego, CA) were used at 1:100 dilution. PE-conjugated H2-Db RAHYNIVTF tetramer recognizing a dominant MHC-I epitope of E7 antigen [27] was a kind gift of Dr. Yvonne Paterson. Rabbit anti-HPV-16 E6 (N-17) and E7 (ED-17) antibodies (Santa Cruz Biotechnology, Santa Cruz, CA) were used at 1:100 dilution.

### Proliferation assays

For mixed leukocyte reactions C57BL/6 DCs were electroporated with TC-1 RNA (50 μg/10^6 ^DCs) or loaded with TC-1 cells killed with UVB; incubated with TNF-α and LPS for 48 h; washed twice with PBS; and subjected to gamma-irradiation (20 Gy). DCs were seeded in 96-well round-bottom plates at various dilutions in RPMI containing 10% FBS. BALB/c spleen lymphocytes were procured as described [28]. Constant number of BALB/c lymphocytes (1 × 10^5 ^cells/well) were incubated with irradiated C57BL/6 DCs at increasing ratios (DC: lymphocytes) for 5 days at 37°C. [^3^H]thymidine (NEN Life Science, Boston, MA, 1 μCi/well) was added for 18 hours at 37°C. Samples were recovered on glass fiber filters and analyzed with a Microbeta Trilux (Perkin Elmer Wallac, Inc., Gaithersburg, MD). Each experimental value was determined three times. For some experiments, splenocytes from vaccinated animals were cultured with gamma-irradiated (20 Gy) tumor cells in the presence of 20 U/ml of recombinant murine IL-2 (Peprotech). After 5 days 1 μCi/well of [^3^H]thymidine was added for 18 hours at 37°C.

### Chemotaxis assay

Migration of DCs towards murine macrophage inflammatory protein (MIP)-3α or MIP-3β (R&D Systems, Minneapolis, MN) was assessed in 96-well chemotaxis chambers using an 8 μm-pore nitrocellulose membrane (Neuroprobe, Gathersburg, MD) [29]. Pyrogen-free RPMI 1640 containing 1% BSA was used as chemotactic media. Results are presented as chemotactic index (CI), defined as fold increase in cell migration in the presence of chemotactic factors compared to chemotactic media alone. Each experiment was performed in triplicate.

### IFN-γ ELISPOT

ELISPOT was performed as previously described [28]. We used purified anti-mouse IFN-γ (R4-6A2) for capture and biotin anti-mouse IFN-γ (XMG1.2) (both BD Pharmingen) for detection.

### Immunostaining

Solid tumor samples (n = 6 for each experimental group) were snap-frozen in ornithine carbamyltransferase (OCT, Tissue Tek, Sakura, Torrance, CA). For immunofluorescense analysis, sections were fixed in cold acetone for 10 minutes and sequentially incubated with anti-mouse CD16/32 antibody (1:100 dilution), FITC-anti-mouse CD3 (17A2) and biotin-anti-mouse CD8 (53–67, 1:100 dilution, both BD Pharmingen). After incubation with streptavidin tetramethylrhodamine conjugate (Molecular Probes, Eugene, OR) (1:200 dilution), sections were counterstained with 4',6'-diamidino-2-phenylindole hydrochloride (DAPI) and inspected under the fluorescent microscope.

For immunofluorescense of DCs following electroporation with TC-1 RNA, electroporated DCs were seeded on glass coverslips and cultured in RPMI in the presence of 10% FBS and 10 ng/ml GM-CSF for two days. Cells were fixed with acetone and consecutively stained with anti-HPV-16 E7 antibody (ED-17, Santa Cruz Biotechnologies) and anti-rabbit FITC (BD Pharmingen). Slides were counterstained with DAPI. Images were acquired through Cool SNAP Pro color digital camera (Media Cybernetics, Carlsbad, CA). Ten different fields for each sample at × 400 magnification were evaluated for cell counting.

### Immunoprecipitation and Western blot

For E7 immunoprecipitation and Western blot analysis, ID8-E6/7 and ID8 control cells were lysed in M-Per mammalian protein extraction kit (Pierce, Rockford, IL) plus protease inhibitor cocktail (HALT Protease inhibitor kit, Pierce). Lysate concentration was determined by the Bradford assay (Biorad; Hercules, CA). Two hundred micrograms of extract was subjected to immunoprecipitation with rabbit anti-HPV16 E7 (ED-17, Santa Cruz Biotechnologies). Immunoconjugates were collected on protein A-agarose (Invitrogen), washed with lysis buffer, and resolved on 15% acrylamide gels. Proteins were transferred to polyvinylidene difluoride membranes (Immobilon-P, Millipore) and probed with mouse anti-E7 antibody (clone 8C9, Zymed Labs Inc., San Fransisco, CA). Sheep anti-mouse IgG-HRP (NA93IV, Amersham Biosciences, Buckinhamshire, UK) was used as the secondary reagent. Proteins were visualized by enhanced chemiluminescence (Lumigen PS-3, Amersham) and exposure to Kodak X-Omat Blue film.

### RT-PCR and Real-Time Quantitative Reverse Transcription-PCR

The expression of E6 and E7 was demonstrated using RT-PCR with the following primers: E6 forward primer (F), 5'-AAA GCA GAC ATT TTA TGC ACC A-3'; E6 reverse primer (R), 5'-TCA TGC AAT GTA GGT GTA TCT CC -3'; E7 F 5'-CAC GTA GAG AAA CCC AGC TGT A -3'; E7 R 5'-GTA CCC TCT TCC CCA TTG TT-3'. The RT portion of the RT-PCR was conducted using SuperScript reverse transcriptase (Invitrogen) at 50°C (10 min). The PCR cycling was conducted with Taq polymerase for both sequences at 94°C (3 min), 50°C (1 min), and 72°C (1 min) for 40 cycles.

The AbiPrism 7700 Sequence Detection System and SYBR green I PCR kits (both Applied Biosystems, Foster City, CA) were used for Real-Time PCR as described previously [30]. The following primers were used: E6: F 5'-GAC TTT GCT TTT CGG GAT TTA TGC -3', R 5'-TCA CAC AAC GGT TTG TTG TAT TGC-3'; E7: F 5'-CTG GAC AAG CAG AAC CGG ACA-3', R 5'-TGC TTT GTA CGC ACA CCG AA-3'. We normalized the cDNA load to mouse glyceraldehyde-3-phosphate dehydrogenase (GAPDH) with primers GAPDH F 5'-CCT GCA CCA CCA ACT GCT TA-3' and GAPDH R 5'-CAT GAG TCC TTC CAC GAT ACC A-3'. Data were expressed as relative units to GAPDH mRNA molecules. Molecules were considered to be present if more than five copies of mRNA were detected for every 10^6 ^copies of GAPDH mRNA.

### Statistical analysis

A two-tailed Student's t-test was applied to determine differences between two groups. For multiple comparisons we performed ANOVA with post-analysis comparisons by the Tukey-Kramer multiple comparisons test. Non-parametric studies were performed by using the Mann-Whitney U test. A value of p < 0.05 was considered significant. Data are expressed as mean ± SD. Data was analyzed using Graph Pad Instat software (GraphPad Software, Inc., San Diego, CA).

## Results

### Preparation of DCs electroporated with TC-1 cell RNA (RNA-DCs)

**Figure 1:**
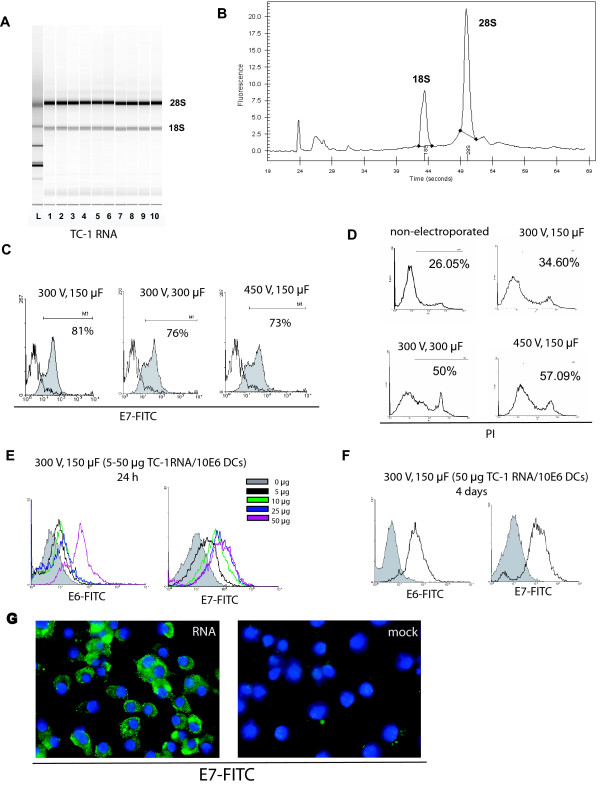
Expression of tumor-associated HPV E6 and E7 antigens by bone marrow-derived DCs after RNA electroporation. (**A**) Gel-like image obtained from 10 independent samples of TC-1 RNA analyzed by the Agilent bioanalyzer. (**B**) Electropherogram of sample 1 from A showing ribosomal RNA peaks. (**C**) Flow cytometry analysis of intracellular E7 expression in CD11c^+ ^cells 24 hours after electroporation with TC-1 RNA at different voltage and capacitance conditions. Electroporation was performed with 25 μg TC-1 RNA/10^6 ^DCs (grey): mock electroporated (white). (**D**) Flow cytometry analysis of DC viability 24 hours after electroporation at different conditions of voltage and capacitance. Electroporation was performed with 25 μg TC-1 RNA/10^6 ^DCs. X-axis reflects propidium iodide (PI) staining. (**E**) Flow cytometry analysis of intracellular E6 and E7 expression in CD11c^+ ^cells 24 hours after electroporation with different amounts of TC-1 RNA at 300V and 150 μF. The experiment was repeated two times with similar results. (**F**) Flow cytometry analysis of intracellular E6 and E7 expression in CD11c^+ ^cells 4 days after electroporation with 50 μg of TC-1 RNA/10^6 ^DCs at 300V and 150 μF. RNA electroporated (white); mock electroporated (grey). (**G**) Immunofluorescence of DCs stained with anti-HPV E7 antibody 24 hours after electroporation with TC-1 cell RNA or mock electroporated. Cells were counterstained with DAPI. 200X magnification. All experiments were repeated twice with similar results.

To test the efficacy of loading DCs with tumor antigen by RNA electroporation we used TC-1 cells, a mouse adenocarcinoma cell line generated by cotransfection of lung epithelial cells with HPV-16 E6 and E7 genes and H-Ras [31], which has been used to test E6 and E7-targeted tumor immunotherapy [32-34]. Reproducible amounts of total RNA ranging between 20–35 μg RNA/10^6 ^cells were obtained from cultured TC-1 cells (Mean = 27.05, SD = 9.183, N = 10 independent samples). High quality RNA was procured (Figure 1A), with rRNA ratio (28S/18S) reproducibly greater than 2.00 (Figure 1B).

To determine the best conditions for RNA electroporation in our system, bone marrow-derived DCs were electroporated with tumor cell RNA using different capacitance and voltage settings and 25 μg RNA per 10^6 ^DCs, which represents RNA from approximately one tumor cell per DC. The reaction was performed in a total volume of 200 μL. As shown in Figure 1C, the highest expression of E7 antigen in live electroporated DCs, was obtained at 300 V and 150 μF. These settings yielded 50% viability in electroporated DCs, as determined by flow cytometry PI exclusion analysis (Figure 1D). Similar viability levels were obtained in DCs electroporated in the absence of RNA (mock, not shown).

To optimize DC electroporation, we electroporated DCs with different amounts of TC-1 RNA (5–50 μg/10^6 ^DCs) using the previously determined settings (300 V, 150 μF). E6-transcripts are longer than E7. As shown in Figure 1E, high E6 expression was observed only after electroporating 50 μg TC-1 RNA/10^6 ^DCs. Higher RNA amounts did not result in increased E6 expression (not shown). Electroporation with 50 μg RNA/10^6 ^DCs also ensured high expression of E7-antigen in DCs (Figure 1E). As shown in Figure 1F, high expression of E6 and E7 protein was detectable by flow cytometry for at least 4 days after RNA electroporation with 50 μg of TC-1 RNA/10^6 ^DCs. Immunofluorescence staining confirmed the presence of E7 protein in the cytoplasm of DCs 24 hours after electroporation with 50 μg TC-1 RNA/10^6 ^DCs (Figure 1G).

### Preparation of DCs pulsed with UV irradiated TC-1 cells (UV-DCs)

**Figure 2:**
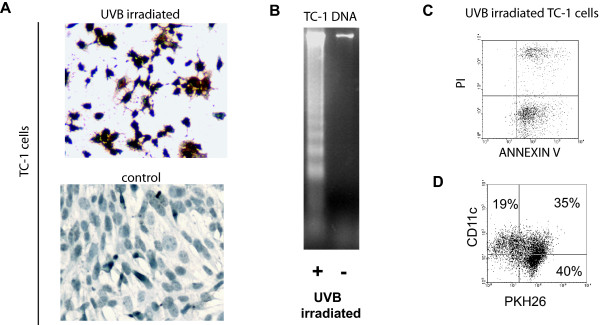
UVB-induced apoptosis of TC-1 cells and maturation of TC-1 antigen-loaded DCs. (**A**) TUNEL assay of UVB-treated and control TC-1 cells 24 hours post-irradiation. (**B**) DNA ladder assay of TC-1 cells 24 hours after irradiation with UVB light. (**C**) Flow cytometry analysis of annexin V in UVB-treated cells 24 hours post-irradiation. (**D**) Percentage of DCs that have engulfed tumor cells, as determined by flow cytometry. DCs were pulsed with cells killed by UVB. Apoptotic tumor cells were labeled with PKH26 prior to pulsing. DCs were stained with antibody against CD11c.

We tested various doses of ultraviolet-B (UVB) light and exposure times to identify UVB conditions that kill greater than 95% of TC-1 cells (not shown). Irradiation with 1500 μW/cm^2 ^UVB for 10 min induced apoptosis in TC-1 cells as assessed by DNA fragmentation detectable by TUNEL assay (Figure 2A) and DNA laddering (Figure 2B), while by flow cytometry the majority of cells were annexin-V positive or propidium iodide and annexin-V double positive within 24 hours (Figure 2C).

To verify the uptake of UV-irradiated cells by DCs, UV-irradiated TC-1 cells were labeled with PKH26 fluorescent dye prior to pulsing of DCs. DCs and UV-irradiated tumor cells were cocultured for 18 hours to allow the uptake of tumor cell material by DCs. Cells were then stained with antibody against CD11c, and CD11c^+ ^DCs were analyzed for PKH26 expression with flow cytometry. More than 60% of DCs had taken up fluorescent tumor cells (Figure 2D), compared with a background level of 1% in the control sample containing DCs and UV-irradiated cells admixed just before analysis (not shown).

### Maturation of RNA-DCs and UV-DCs

**Figure 3:**
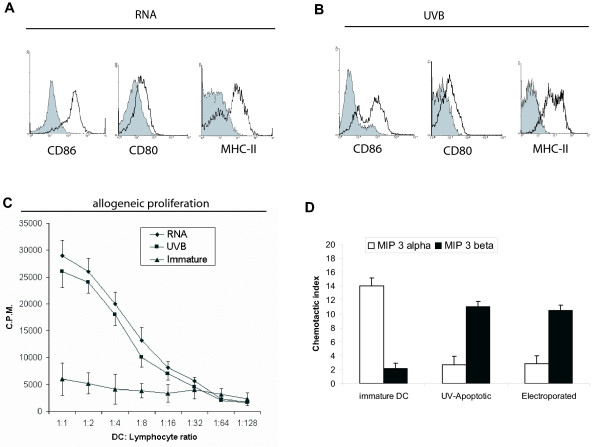
(**A**) Expression of maturation markers in E7^+^CD11c^+ ^gated cells 72 hours after electroporation with TC-1 RNA (50 μg/10^6 ^cells). LPS and TNF-α were added to the culture medium 24 hours after electroporation. (white): specific antibody; (grey): isotype control. The experiment was repeated two times with similar results. (**B**) Expression of markers in CD11c^+ ^gated cells 72 hours after phagocytosis of UVB-irradiated TC-1 cells. LPS and TNF-α were added to the culture medium after 24 hours of coincubation. (white): specific antibody; (grey): isotype control. (**C**) Proliferation of allogeneic BALB/c splenocytes induced by C57BL/6 DCs electroporated with TC-1 cell RNA (RNA) or pulsed with UV-irradiated TC-1 cells (UVB). DCs were treated with TNF-α and LPS. Data were obtained 48 hours after stimulation of splenocytes and are expressed as the mean ± SEM of two experiments with triplicate observations per experiment. (**D**) Migration of DCs towards MIP-3α or MIP-3β 72 hours after electroporation with TC-1 RNA (RNA) or pulsing with UV-irradiated TC-1 cells (UVB). LPS and TNF-α were added to the culture medium after 24 hours of coincubation. Chemotactic index is defined as the fold increase in cell migration caused by the chemotactic factors. Data are expressed as the mean ± SD of two experiments with quadruplicate observations per experiment.

We assessed whether DCs prepared by RNA electroporation or pulsing with UV-irradiated tumor cells respond differently to maturation stimuli such as TNF-α and LPS. Significant surface expression of CD86 and CD80 and MHC-II molecules was noted in DCs 48 hours post-electroporation with tumor cell RNA, as described above, in the presence of TNF-α and LPS (Figure 3A). Similarly, immature DCs incubated with TC-1 cells exposed 24 hour earlier to lethal dose of UVB radiation upregulated CD80, CD86 and MHC-II 48 hours post-phagocytosis, in the presence of TNF-α and LPS (Figure 3B).

Next, we assessed whether DCs electroporated with tumor cell RNA (RNA-DCs) or pulsed with UV-irradiated tumor cells (UV-DCs) and matured with TNF-α and LPS differ in vitro in their ability to induce proliferation of allogeneic lymphocytes or to migrate towards lymphoid organ chemokines. Immature DCs were incubated for 48 with TC-1 cells exposed 24 hours earlier to lethal UVB radiation or were electroporated with tumor cell RNA, as described above. To minimize differences in the amount of original tumor antigen used with both loading procedures, DCs were incubated with UVB-irradiated cells at 1:2 ratio (DC, tumor cells) or electroporated with an equivalent amount of total RNA (50 μg RNA corresponding to 2 × 10^6 ^tumor cells per 10^6 ^DCs). RNA-DCs stimulated proliferation of allogeneic spleen lymphocytes in a similar manner to UV-DCs. As expected, unpulsed immature DCs did not stimulate allogeneic reaction (Figure 3C).

A switch in chemokine receptors is a hallmark of DC maturation. Among others, this entails upregulation of CCR7 and downregulation of CCR6 [35]. RNA-DCs and UV-DCs matured with TNF-α and LPS exhibited similar migratory properties. As shown in Figure 3D, both DCs lost the ability to migrate towards macrophage inflammatory protein 3-alpha (MIP3-α), the ligand for CCR6, and acquired the ability to migrate towards MIP-3β, the ligand for CCR7.

### Development of additional tumor targets expressing HPV-16 E6 and E7

**Figure 4:**
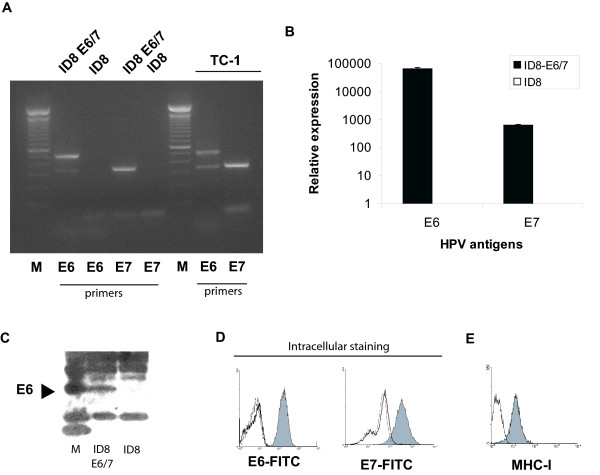
Expression of HPV-16 E6 and E7 antigens in retrovirus-transduced ID8 cells. (**A**) RT-PCR analysis for HPV-16 E6 and E7 transcripts in ID8 cells transduced with HPV-16 E6 and E7 genes; ID8 cells transduced with empty vector (negative control); and TC-1 cells (positive control). M: molecular markers. (**B**) Real-time quantitative PCR analysis of E6 and E7 transcripts in ID8 cells transduced with HPV-16 E6 and E7 genes and ID8 cells carrying empty vector. (**C**) Western blot analysis of cell lysate showing presence of E6 protein in samples obtained from ID8 E6/7 cells but not in ID8 cells carrying empty vector. M: molecular markers. (**D**) Flow cytometry analysis of intracellular E6 and E7 proteins in ID8 cells transduced with HPV-16 E6 and E7 genes. (grey): ID8 E6/7 cells; (white): ID8 cells transduced with empty vector, (dotted line): isotype control. (E) Expression of MHC-I by ID8-E6/7 cells. (grey): ID8 E6/7 cells; (white): isotype control.

To further compare tumor vaccination with RNA-DCs and UV-DCs, we engineered an additional tumor cell line expressing HPV E6 and E7. We used the syngeneic C57BL/6 murine ovarian cancer cell line ID8. ID8 cells transduced with the retroviral vector LXSN16E6E7, which encodes the HPV16 E6 and E7 genes, as well as the neomycin phosphotransferase gene (ID8-E6/7) stably expressed E6 and E7 mRNAs under genetecin pressure, compared to ID8 cells transduced with the empty vector (ID8) (Figure 4A and 4B). Immunoprecipitation followed by Western blot analysis revealed the presence of E6 protein in ID8-E6/7 cells but not in ID8 cells transduced with empty vector (Figure 4C). Moreover, intracellular staining of ID8 E6/7 cells showed the presence of HPV-16 E6 and E7 proteins (Figure 4D). As the parental ID8 cell line [36], ID8-E6/7 cell line expressed MHC class-I molecules (Figure 4E).

### RNA-DCs and UV-DCs are immunogenic in vivo

We compared the efficacy of RNA-DCs and UV-DCs to induce tumor vaccination. To minimize differences in the amount of tumor antigen between pulsing procedures, DCs were incubated with UV-irradiated cells at 1:2 ratio (DC, tumor cell) or electroporated with an amount of total RNA equivalent to two tumor cells per DC, as above. DCs were matured with TNF-α and LPS. Mock DCs were prepared by electroporation in the absence of tumor RNA followed by maturation with TNF-α and LPS. Healthy animals were vaccinated with RNA-DCs or UV-DCs, while control animals were vaccinated with mock DCs or left unvaccinated (naïve). Splenocytes isolated from the above animals were tested for reactivity against E6 and E7 antigens by incubating them with apoptotic TC-1, ID8-E6/7, ID8 or L-929 cells.

**Figure 5:**
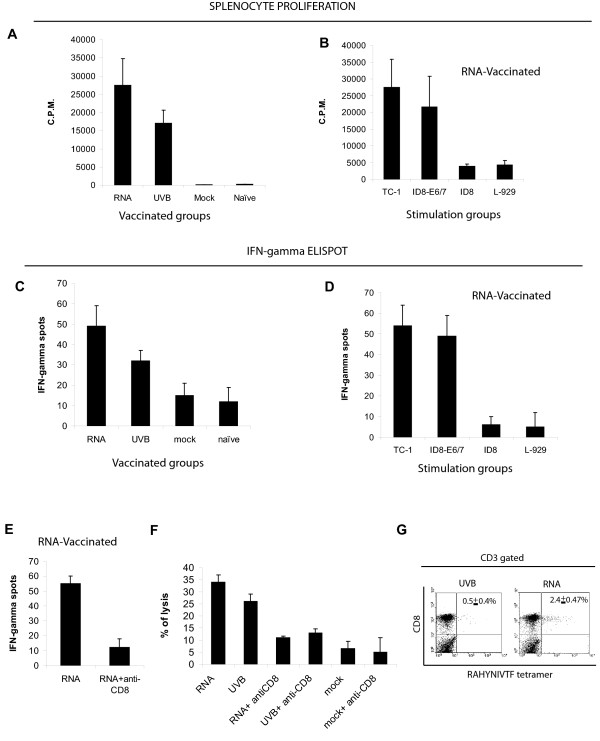
Antitumor immune response in vaccinated animals. (**A**) Proliferation of splenocytes from animals vaccinated with DCs electroporated with TC-1 RNA (RNA); pulsed with UV-irradiated TC-1 cells (UVB); mock-electroporated DCs (mock), or naïve animals. Splenocytes were stimulated in vitro with gamma-irradiated TC-1 cells for 5 days. Data are expressed as the mean ± SD (n = 5). RNA vs. UVB: p < 0.01; RNA vs. mock: p < 0.001; RNA vs. naive: p < 0.001; UVB vs. mock: p < 0.001; UVB vs. naive: p < 0.001 (ANOVA, Tukey-Kramer multicomparison post-test). (**B**) Proliferation of splenocytes from animals vaccinated with DCs electroporated with TC-1 cell RNA. Splenocytes were stimulated in vitro for 5 days with gamma-irradiated TC-1 cells, ID8-E6/E7 cells, ID8 cells carrying empty vector or L-929 cells. Data are expressed as the mean ± SD (n = 5). TC-1 vs. ID8-E6/7: non significant (NS); TC-1 vs. ID8: p < 0.001; TC-1 vs. L-929: p < 0.001; ID8-E6/7 vs. ID8: p < 0.001; ID8-E6/7 vs. L-929: p < 0.001. The experiment was repeated two times with similar results. (**C**) Quantification of IFN-γ producing tumor-reactive splenocytes by ELISPOT from the same experimental groups as in A. Data are expressed as the mean ± SD (n = 5). RNA vs. UVB: p < 0.05; RNA vs. mock: p < 0.005; RNA vs. naive: p < 0.005; UVB vs. mock: p < 0.005; UVB vs. naive: p < 0.005. (**D**) Quantification of IFN-γ producing tumor-reactive splenocytes by ELISPOT from the same experimental groups as in B. Data are expressed as the mean ± SD (n = 5). TC-1 vs. ID8-E6/7: non significant (NS); TC-1 vs. ID8: p < 0.001; TC-1 vs. L-929: p < 0.001; ID8-E6/7 vs. ID8: p < 0.001; ID8-E6/7 vs. L-929: p < 0.001. (**E**) IFN-γ producing cells from splenocytes of RNA vaccinated animals upon CD8 immunodepletion. p < 0.01. (**F**) Cytotoxic assay using TC-1 cells as targets. Tumor cells were incubated at 3:1 effector:target ratio with gradient-purified pooled splenic lymphocytes from animals vaccinated with DCs electroporated with TC-1 cell RNA (RNA) or pulsed with UV-irradiated TC-1 cells (UVB). Some groups were immunodepleted of CD8^+ ^cells. Splenocytes were stimulated in vitro with gamma-irradiated TC-1 cells for 5 days. RNA vs. UVB: p < 0.05; Student's t-test. The experiment was repeated two times with similar results. (**G**) Frequency of anti-E7 CD8^+ ^lymphocytes following DC vaccination. E7 tetramer-positive, CD3-gated CD8^+ ^precursors were quantified in splenocytes from animals vaccinated with DCs pulsed with UVB-irradiated TC-1 cells (UVB) or DCs electroporated with TC-1 cell RNA (RNA), p < 0.05.

When incubated with TC-1 cells, splenocytes from animals vaccinated with RNA-DCs proliferated significantly more than splenocytes from animals vaccinated with UV-DCs (Figure 5A). No proliferation was detected in lymphocytes from animals vaccinated with mock-electroporated DCs or naïve animals (Figure 5A). Similar proliferation was detected when splenocytes from mice vaccinated with RNA-DCs were incubated with TC-1 or ID8-E6/7 cells (Figure 5B), but no proliferation was detected against control ID8 cells lacking E6 or E7, or L-929 cells. This shows that the presence of HPV antigens was critical for T cell proliferation (Figure 5B). Splenocytes from all four groups of animals showed similar proliferative response when stimulated with phytohemagglutinin (not shown), indicating no functional impairment.

We evaluated the frequency of tumor-reactive T cells among splenocytes in each group of animals by IFN-γ ELISPOT analysis. We used ID8-E6/E7 cells as target cells. A significantly higher frequency of tumor-reactive IFN-γ producing cells was found in spleens from animals vaccinated with RNA-DCs compared to animals vaccinated with UV-DCs. No response was observed in splenocytes from mock vaccinated or naïve animals (Figure 5C). Similar response was seen in splenocytes from mice vaccinated with RNA-DCs incubated with TC-1 or ID8-E6/7 cells, while no response was observed against ID8 pr L-929 cells (Figure 5D). Moreover, in the RNA vaccinated group, IFN-γ producing cells were mainly CD8^+ ^cells, since immunodepletion of CD8^+ ^cells in vivo decreased the number of IFN-γ producing cells among isolated splenocytes (Figure 5E).

Higher levels of cytotoxic lymphocyte activity were detected in lymphocytes obtained from animals vaccinated with RNA-DCs relative to the UV-DCs group (Figure 5F). Moreover, CTL activity of splenocytes was abrogated to control levels by immunodepletion of CD8^+ ^cells in vivo (Figure 5F). No lytic activity was observed when control ID8 cells expressing no E6 or E7, or L-929 cells were used as target cells, confirming the specificity of the reaction (not shown).

TC-1 tumors express HPV E6 and E7, offering the opportunity to quantify T cell responses against tumor-associated HPV epitopes through well-characterized tetramers [37]. We compared the ability of RNA-DCs and UV-DCs to generate T cell response against the H2-D^b^-restricted HPV E7 epitope RAHYNIVTF [34]. A four-fold higher frequency of tetramer-positive CD3^+ ^CD8^+ ^cells was detected in splenocytes from animals vaccinated with RNA-DCs compared to animals vaccinated with UV-DCs (Figure 5G).

### Vaccination with RNA-DCs or UV-DCs

**Figure 6:**
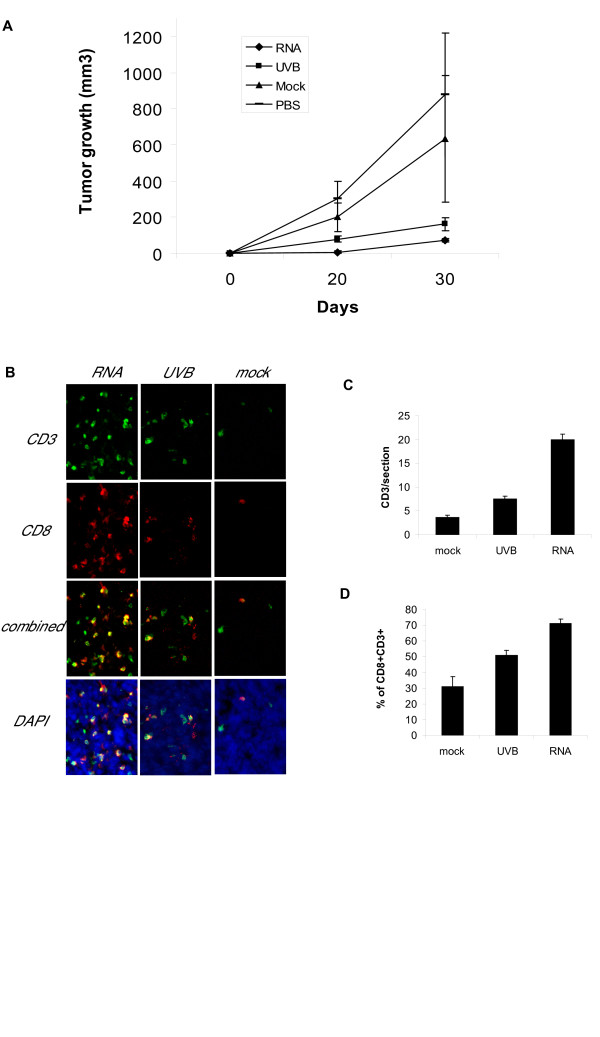
Antitumor efficacy of RNA electroporated DCs. (**A**) Growth of flank TC-1 tumors in animals previously vaccinated with DCs electroporated with TC-1 cell RNA, UV-irradiated TC-1 cells, mock DCs or non-treated. Animals were challenged by injecting 2 × 10^4 ^tumor cells. (n = 5 each group). Data are representative of three independent experiments with similar results. Day 30: RNA vs. UVB: p < 0.01; RNA vs. PBS: p < 0.01; RNA vs mock: p < 0.01; UVB vs. PBS: p < 0.01; UVB vs. mock: p < 0.01; PBS vs. mock: non significant. Mann-Whitney test. (**B**) CD3 and CD8 staining of tumors obtained from animals vaccinated with DCs electroporated with TC-1 cell RNA (RNA), DCs pulsed with UVB-irradiated TC-1 cells (UVB), or mock electroporated DCs. Magnification: 200X. (**C**) Counting of CD3 cells infiltrating TC-1 tumors. Experimental groups as in A. RNA vs. UVB: p < 0.001; RNA vs. mock: p < 0.001; UVB vs. mock: p < 0.001. Kruskal-Wallis non parametric ANOVA test. Dunn's multiple comparisons test. (**D**) Percentage of CD8^+ ^among CD3^+ ^cells infiltrating TC-1 tumors. Experimental groups as in A. RNA vs. UVB: p < 0.05; RNA vs. mock: p < 0.001; UVB vs. mock: NS (Kruskal-Wallis non parametric ANOVA test. Dunn's multiple comparisons test).

To test the efficacy of RNA-DCs and UV-DCs in vivo, healthy mice were vaccinated with three injections of RNA-DCs or UV-DCs prepared as above and administered one week apart. Seven days after the last DC vaccination, animals were challenged with flank subcutaneous TC-1 tumors. Tumor growth was significantly delayed in animals vaccinated with RNA-DCs as well as animals vaccinated with UV-DCs (Figure 6A). Although the difference in tumor growth between mice vaccinated with RNA-DCs and mice vaccinated with UV-DCs was not large, tumors were significantly smaller in mice vaccinated with RNA-DCs. Similar results were obtained in three independent experiments.

Tumors from mice vaccinated with RNA-DCs as well as from animals vaccinated with UV-DCs exhibited significantly higher frequency of CD3^+ ^tumor-infiltrating cells relative to mice vaccinated with mock DCs. A higher frequency of CD3^+ ^tumor-infiltrating cells was detected in animals vaccinated with RNA-DCs compared to animals vaccinated with UV-DCs (Figure 6B, C). Finally, a higher proportion of CD3^+ ^cells were CD8^+ ^in animals vaccinated with RNA-DCs compared to animals vaccinated with UV-DCs or mock DCs (Figure 6D).

## Discussion

The present work addressed the relative efficacy of tumor vaccines prepared with DCs either electroporated with tumor RNA or with dead tumor cells. We used total RNA from tumor cells isolated with a common laboratory method and UVB irradiated tumor cells and optimized the conditions to minimize difference in amount of tumor cell material used to pulse DCs. We used the E7 MHC-I-restricted epitope RAHYNIVTF to quantify the intensity of CD8^+ ^T cell response against tumor-associated antigens. The present results show that electroporation with whole tumor cell RNA and pulsing with UVB-irradiated tumor cells are both effective in eliciting antitumor immune response, but RNA electroporation results in more potent tumor vaccination. The efficacy of the vaccination by RNA-electroporated DCs was dependent on the presence of CD8^+ ^cells, since in vivo depletion of these cells abrogated the reported effect. Importantly, vaccination with RNA-electroporated DCs expressing E6 and E7 significantly enhanced infiltration of CD3^+^CD8^+ ^into the tumors.

To our knowledge this is the first direct comparison between irradiated cells and whole RNA as a source of whole tumor antigen to prepare DC based tumor vaccines. Because the same number of cells was used to derive tumor antigen with both methodologies, the above findings indicate that RNA electroporation is an efficient methodology for loading DCs with tumor antigen. A previous report also found that apoptotic tumor cell pulsing is not an efficient approach to tumor vaccination [38]. Several reasons may account for our findings. First, antigen up-take may be less efficient with pulsing dying cells compared to RNA electroporation. Since UVB irradiation has been shown to result in a mixed population of apoptotic and necrotic tumor cells [16], it is possible that either process leads to degradation of important antigens [39], resulting in suboptimal antigen processing or presentation. Second, apoptotic DNA may bind to MHC class molecules and interfere with antigen presentation [40]. Third, although non-viral methods of DNA transfection of DCs are inefficient, the efficiency of gene transfer with RNA electroporation resembles that of transfection with recombinant viruses [41]. Finally, another advantage of RNA transfer of DC over pulsing DC with protein antigens is that endogenously synthesized antigens have better access to the class I MHC pathway [42].

It is noteworthy to comment that different maturation protocols can modify the capability of DCs to effectively present antigens upon RNA electroporation. In a series of elegant studies, Zobylawski *et al*., have shown that human DCs treated with a maturation cocktail formulated with TNF, IL-1β, IFN-γ, prostaglandin E2, and the Toll like receptor (TLR) 8 agonist R848 were able to generate an efficient immune response upon RNA electroporation; while addition of the TLR3 ligand poly(I:C) to the maturation cocktail rendered DCs unable to express proteins from electroporated RNA [43]. Indeed, the formulation of appropriate maturation cocktails is one of the challenges that faces the generation of effective DC-based vaccines for clinical use [44]. Thus, the use of different activation protocols might have produced different results in our studies. Further, since in our studies we used murine bone marrow-derived DCs, they may not directly translate to human monocyte-derived dendritic cells used for clinical studies. Additional studies with human monocyte-derived dendritic cells must be performed in order to determine the clinical relevance of our findings. Finally, it should be noted that our work compared RNA to dead tumor cells and our findings on superiority of RNA as a source of whole tumor antigen may not be relevant to alternate methods of preparing whole tumor cell protein, such as tumor lysates [45], which may preserve tumor antigens or do not interfere with antigen presentation.

## Conclusion

Collectively, our data suggest that electroporation of whole tumor RNA represents a direct and effective way of delivering tumor antigen to DCs ex vivo. Coupled with the easier procurement of tumor RNA compared to the generation of tumor cell lines, these findings suggest that RNA electroporation should be a preferred method of loading DCs with whole tumor antigen in clinical trials.

## Abbreviations

DCs: dendritic cells; DEPC: diethylpyrocarbonate; FACS: fluorescence activated cell sorting; GM-CSF: granulocyte macrophage-colony stimulation factor; HPV: human papilloma virus; IFN: interferon; LPS: lipopolysaccharide; MHC: major histocompatibility complex; MIP: macrophage inflammatory protein; PBS: phosphate buffered saline; RNA: ribonucleic acid; RPMI: Rosewell Park Memorial Institute – cell culture medium; TLR: Toll-like receptor; TNF: tumor necrosis factor; UVB: ultraviolet-B

## Competing interests

The authors declare that they have no competing interests.

## Authors' contributions

FB participated in study design, carried out the in vitro and in vivo studies and drafted the manuscript and figures. MCC assisted with all the in vitro and in vivo experiments. GC conceived of the study, participated in its design and coordination, and finalized the manuscript.
